# Effect of Print Orientation on Sintering Shrinkage of BMD 316L Stainless Steel: An Empirical and Numerical Study

**DOI:** 10.3390/ma19153294

**Published:** 2026-08-03

**Authors:** Ayechew Aklilu, John Belding, Joe Strauss, Brett D. Ellis

**Affiliations:** 1 Mechanical Engineering, University of Maine, 75 Long Road, Orono, ME 04469, USA; ayechew.aklilu@maine.edu; 2 Advanced Manufacturing Center, University of Maine, 5796 AMC Building, Orono, ME 04469, USA; 3 HJE Company, 820 Quaker Road, Queensbury, NY 12804, USA; 4 Mechanical Engineering Technology, University of Maine, 5711 Boardman Hall, Orono, ME 04469, USA

**Keywords:** Bound Metal Deposition (BMD), sintering simulation, finite element modeling, particle swarm optimization

## Abstract

**Highlights:**

- As-printed orientation-dependent shrinkages of 316L BMD specimens are measured via dilatometer.
- A calibrated thermo-elastic-plastic model explains the observed shrinkages.
- Print orientation may influence differential shrinkage beyond sintering orientation alone.

**Abstract:**

This study empirically and numerically investigates sintering-induced shrinkage of 316L stainless steel cylinders fabricated via Bound Metal Deposition (BMD). Sintering shrinkages were measured for three cylindrical specimens, one each in *x*-, *y*-, and *z*-print orientations, via a dilatometer. Each specimen was horizontally sintered to 1360 °C via a prescribed temperature profile to maintain identical sintering conditions between specimens. Numerically, a two-dimensional axisymmetric finite element model was implemented in Abaqus using a user-defined viscoplastic constitutive model incorporating porosity- and temperature-dependent viscosities, grain-growth kinetics, and capillary-driven sintering stress. Model parameters were calibrated using particle swarm optimization by minimizing a combined error metric for temperature-dependent shrinkage responses and final relative densities. Results indicate: (1) final shrinkages of 13.5%, 13.7%, and 14.3% for the *x*-, *y*-, and *z*-oriented specimens, respectively, and (2) the optimized numerical model utilizing literature-informed material property constraints results in shrinkages and final densities that are in good agreement with empirical observations. Importantly, this work (1) suggests sintering shrinkage may depend on print orientation, in addition to furnace orientation, for BMD-fabricated 316L stainless steel, and (2) adds an initial dilatometer data set for BMD 316L to the literature. More broadly, this work supports future design activities via an experimental and numerical characterization of sintering behavior.

## 1. Introduction

Additive Manufacturing (AM), commonly known as 3D printing, is a manufacturing process that fabricates components layer by layer, placing material only where necessary. This approach enables exceptional design flexibility and reduces material waste. Originally developed as a rapid prototyping tool for plastics, AM has evolved into a robust manufacturing method suitable for a wide range of materials, including metals [1].

Among the various metal AM technologies, Powder Bed Fusion (PBF) methods such as Selective Laser Sintering (SLS) and Electron Beam Melting (EBM) utilize high-energy laser and electron beams, respectively, to fuse or melt metal powders into solid, three-dimensional parts. Although these processes can produce parts with excellent mechanical properties, they typically require significant capital investment and pose challenges related to powder handling and material cross-contamination [2,3].

In contrast to beam-based powder fusion methods, extrusion-based approaches such as Bound Metal Deposition (BMD) offer a more accessible alternative. The first BMD manufacturing step extrudes feedstock composed of metal powder embedded within a wax–polymer binder through a heated nozzle to form parts layer by layer [4]. This extrusion behavior is enabled by the thermal characteristics of the feedstock material, specifically the binder’s softening and melting transitions and accompanying changes in viscosity, which permit flow through the nozzle while avoiding thermal degradation [5]. BMD feedstocks typically contain 50–60% metal powder by volume, with the remainder consisting of a wax–polymer binder system [6,7]. The relatively low melting point of the binder compared with the metal powder allows extrusion at moderate temperatures around 175 °C for first-generation (v1) and 165 °C for second-generation (v.2) materials [8].

After printing, v.2 green-state parts enter a thermal cycle in which the polymer binder is removed by binder burnout (i.e., thermal decomposition and evaporation), yielding a porous brown-state part. The brown-state part remains in the furnace, which is purged and heated to a sintering temperature just below the alloy’s melting point, at which solid-state diffusion fuses the particles together and reduces porosity. After sintering, the relative density approaches 0.96 to 0.99 of the theoretical density with residual porosity of approximately 1 to 4%, corresponding to an isotropic linear shrinkage of about 16% [9]. The result is a dense, polymer-free metal with near-isotropic properties and minimal evidence of the original powder morphology [10].

Sintering is a solid-state bonding process in which loose metal, ceramic, or other powdered materials are fused into a relatively dense structure through controlled heating. The process occurs at temperatures slightly less than the melting point of the primary constituents and is driven by the system’s tendency to reduce its total energy, particularly surface energy.

Phenomenologically, sintering proceeds through three stages [11,12]. In the initial stage, interparticle necks form and grow with minimal dimensional change (e.g., linear shrinkage ≤ 3% [11,13]). For loose spheres, bulk relative density is typically ≤70% of the theoretical density [12], particle size is approximately the initial particle size, and the surface area remains ≥50% of the original [11]. In the intermediate stage, pores round and elongate yet remain connected to the surface. Densification becomes significant and grain growth initiates late in this stage and bulk relative density increases to 70–92% of the theoretical density [11,12]. In the final stage, pores close and become isolated. Densification slows while coarsening dominates, and total porosity decreases to ≤8% [11,12]. At the microstructural level, sintering is governed by diffusional and viscoplastic transport with distinct atomic and pore migration pathways, including non-densifying surface diffusion, grain-boundary diffusion, lattice diffusion, evaporation–condensation, and stress-assisted viscous creep flow [11,14]. Together, these mechanisms drive densification and microstructural evolution during sintering by redistributing material at different structural levels [11,15].

To understand and predict these processes, sintering has been modeled at three different scales: microscopic, mesoscopic, and macroscopic. Microscopic models, such as the two-sphere model [16,17], represent sintering at the particle scale and describe the process in three stages: initial neck formation, shape evolution with interconnected porosity, and final pore isolation [11,16]. At the intermediate length scale, mesoscopic models use simplified geometries to simulate mass transport and microstructural evolution, which influence material properties like strength and conductivity [18]. Examples include phase-field models [19] and cellular automata [20]. At the coarsest length scale, macroscopic models focus on predicting the overall shrinkage and deformation of industrial components during sintering. They rely on continuum mechanics and are especially valuable for process optimization and mold design in ceramic and powder metallurgy applications [21,22].

To describe the complex viscoplastic behavior of materials during sintering, a variety of constitutive frameworks have been developed. Among them, the continuum theory of sintering formulated by Olevsky [21] has become especially influential, providing a practical framework that relates densification kinetics to porosity and effective viscosity in a manner well suited for finite element implementation. Building upon Skorohod [23], Olevsky [21] established a continuum approach to viscous flow in porous bodies. Building on this foundation, Reiterer et al. [24] advanced the Skorohod–Olevsky viscous sintering (SOVS) model by introducing an Arrhenius-type viscosity law in place of the earlier polynomial form and implementing it in JAS3D. This modification yielded sigmoidal densification curves in closer agreement with experiments, captured heating-rate sensitivity with a single activation energy, and provided a bridge to Master Sintering Curve analysis [24]. Arguello et al. [25] further verified and validated the nonlinear finite-element implementation of the SOVS model, testing it against ZnO bilayer and concentric-cylinder experiments and analyzing numerical performance. Their work revealed the need for fine meshes to capture curvature effects and showed incremental improvements when coupling Arrhenius viscosity with kinetic Monte-Carlo (KMC)-derived porosity laws and grain-growth corrections. More recently, Petrović et al. [26] emphasized the role of thermal effects by integrating transient heat transfer into modified SOVS simulations, demonstrating that temperature non-uniformity, especially in larger components can induce significant local densification gradients, shape distortions, and residual stresses, making uniform-temperature assumptions unreliable for large, geometrically complex parts.

While the Olevsky framework emphasizes a continuum constitutive description suitable for simulation, Ashby [27] approached the problem from a mechanistic perspective by developing sintering diagrams that unify multiple transport processes into a single map. These diagrams distinguish between mechanisms that merely promote neck growth and those that actually reduce porosity, thereby clarifying the conditions under which densification can occur. The contrast is clear: Ashby’s diagrams serve primarily as a materials-science tool for identifying dominant mechanisms and guiding process selection, whereas Olevsky’s model provides a continuum mechanics law for directly simulating shrinkage and stress evolution. Building on such mechanistic insights and continuum formulations, Kraft and Riedel [28] presented a comprehensive finite element sintering model that incorporated grain growth, pore evolution, and transitions from open to closed porosity. Applied to silicon carbide compacts, their simulations demonstrated how variations in initial green density strongly influence shrinkage, warpage, and residual porosity, thereby showcasing the power of integrated finite element models of compaction and sintering to replace trial-and-error process design with predictive optimization.

Building on these developments, this study adopts Olevsky’s viscoplastic continuum framework as the constitutive basis, with bulk viscosity reformulated using the power-law relation proposed by Hsueh et al. [29]. Although the Abouaf et al. [30] critical-porosity expression was considered for comparison, Hsueh’s formulation was selected because of its demonstrated reliability in predicting the densification of binder-jet 316L stainless steel [31]. To implement this, a user-defined CREEP subroutine was developed in Abaqus 2023 and coupled with a 2D axisymmetric finite element model of cylindrical 316L BMD specimens. Complementary dilatometry experiments provided shrinkage data under controlled thermal cycles. Model parameters were calibrated using particle swarm optimization (PSO), by minimizing a combined error metric between measured and simulated temperature-dependent shrinkage curves and final relative densities. This study has two primary objectives. First, it presents experimental shrinkage measurements and develops and calibrates a numerical model based on established constitutive formulations to fit and interpret the observed behavior. Second, by sintering directionally printed specimens under identical conditions, the study considers how print orientation may contribute to differential shrinkage beyond the effect of sintering orientation alone.

## 2. Materials and Methods

### 2.1. Overall Workflow

**Figure 1:**
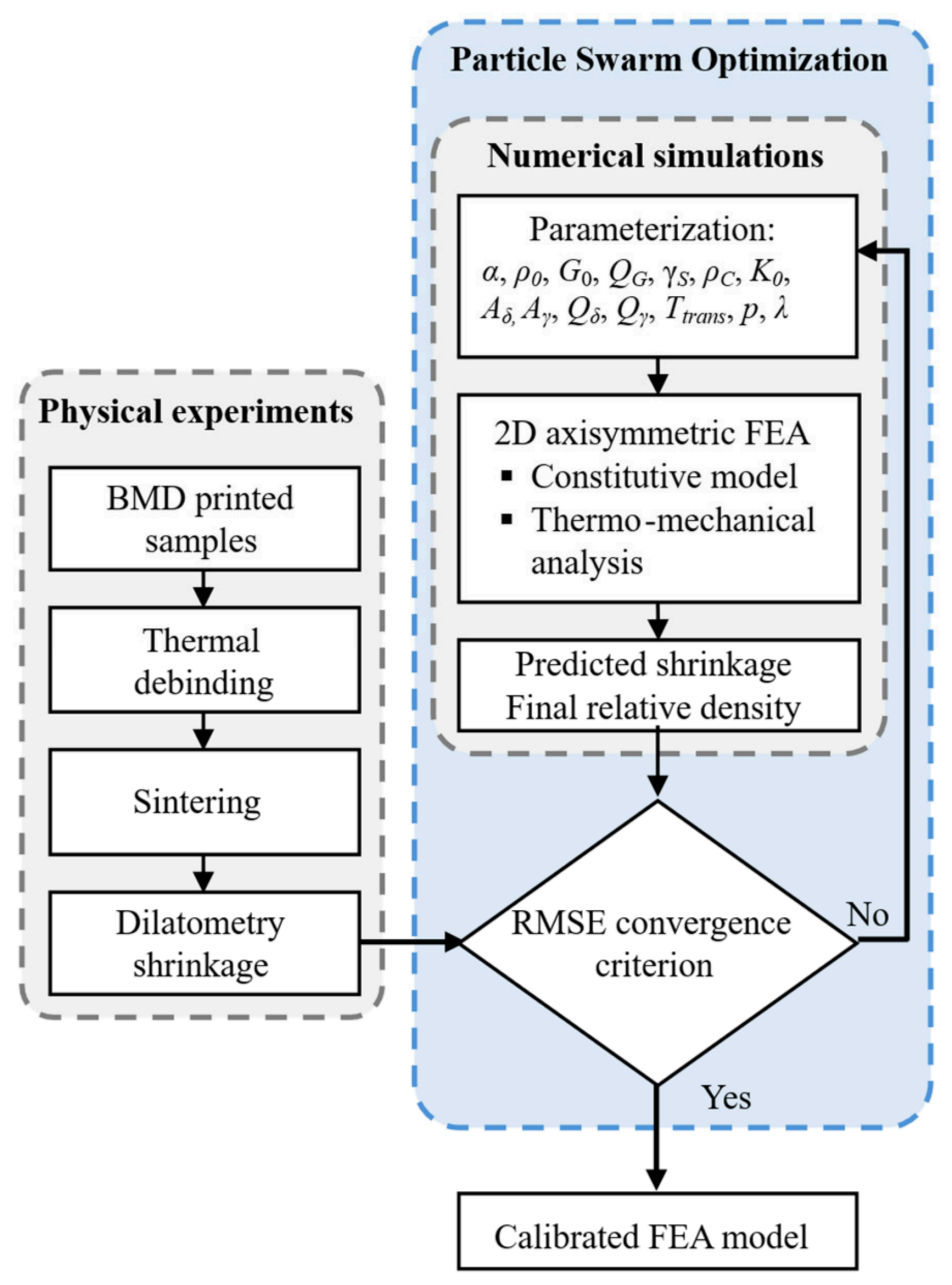
Integrated experimental–numerical workflow for sintering shrinkage modeling and parameter calibration.

As shown in Figure 1, the overall workflow incorporated physical experiments, numerical simulations, and a particle swarm optimization algorithm.

### 2.2. Experimental Methods

A 7.64-mm-diameter by 16.98-mm-long cylindrical specimen geometry was drawn in SolidWorks 2022 (Dassault Systèmes, Vélizy-Villacoublay, France). Specimen geometry was imported into Fabricate v3.3.2 slicing software (Desktop Metal, Burlington, MA, USA), which was utilized to slice three specimen orientations: *x*, *y*, and *z*. For the *x*- and *y*-orientations, each cylinder’s longitudinal axis was in the build plane; for the *z*-orientation, the cylinder’s longitudinal axis was in the build direction.

To account for shrinkage during sintering, Fabricate applies planar orthotropic scale factors of 1.1758 in the build plane (i.e., in the *x*–*y* plane) and 1.1776 in the build direction for 316L materials, meaning as-printed parts are larger than the desired, as-sintered 7.64-mm diameter and 16.98-mm length. For example, as-printed *z*-oriented specimens nominally had 8.98-mm diameters (i.e., 7.64 mm × 1.1758) and were 20.00 mm long (i.e., 16.98 mm × 1.1776). Each cylindrical specimen was sliced to have 4.4-mm-thick walls and solid infill, resulting in a relatively dense, non-porous mesostructure. Each specimen was printed on its own raft (i.e., a printed support platform having a relatively open, porous mesostructured) using Desktop Metal 316L v.2 material.

**Figure 2:**
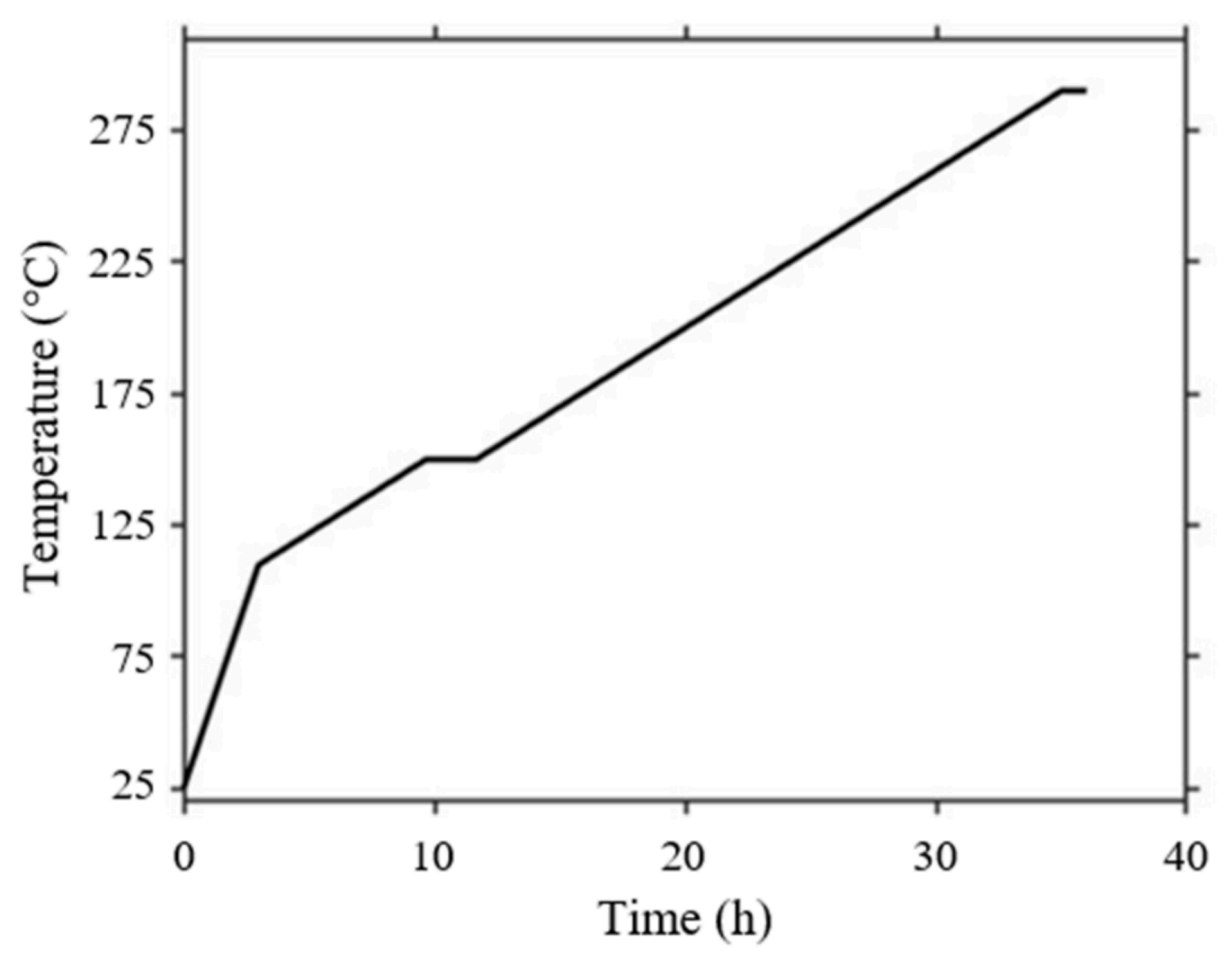
Air-debinding temperature profile used for binder removal.

Although debinding and sintering BMD steps are typically completed within a single temperature cycle in a sintering furnace, debinding and sintering were decoupled in this work to measure linear displacement as a function of temperature and time during sintering via a dilatometer. To prevent the binder from contaminating the dilatometer, debinding was performed in an air furnace (HJE Company, Inc., Queensbury, NY, USA) for a total of 36 h. As shown in Figure 2, the debinding cycle heated the specimen from room temperature to 110 °C at 0.5 °C/min, from 110 °C to 150 °C at 0.1 °C/min, held at 150 °C for 2 h, heated from 150 °C to 290 °C at 0.1 °C/min, and held at 290 °C for 1 h. After the 1-h hold at 290 °C, the oven was de-energized, and the specimens were returned to room temperature at an uncontrolled and unmeasured rate. Relatively slow low-temperature ramp rates (e.g., 0.1 °C/min) were employed to facilitate gradual diffusion and evaporation of paraffin wax, thereby minimizing the risk of distortion.

The air furnace debinding profile shown in Figure 2 was selected based upon: (1) thermogravimetric analysis (TGA) trials of relatively porous rafts to determine suitable debinding environments and temperatures, and (2) debinding trials of cylindrical specimens to determine if relatively dense specimens could survive debinding without slumping. First, two TGA trials were performed—one in an H_2_ environment, and one in an air environment—from room temperature to 600 °C at 0.5 °C/min. In the H_2_ environment, the TGA measured a 2.6% maximum specimen mass loss occurring at approximately 500 °C. In the air environment, the TGA measured a 2.5% maximum mass loss occurring at approximately 315 °C, and a subsequent 0.5% increase in mass (likely due to oxidation) between 315 °C and 600 °C. Next, cylindrical specimens were subjected to debinding trials using the same temperature profile as the TGA temperature profile. The relatively dense, non-porous specimen slumped in the H_2_ environment but maintained its geometry in the air environment. Thus, an air debinding environment with a 300 °C maximum temperature was utilized. Because the production BMD system debinds and sinters in a 97.2% argon–2.8% hydrogen environment in a single thermal process [7], the shrinkage responses reported in this study should be interpreted as a laboratory debind–sinter process, not a direct replication of the full commercial BMD thermal cycle. Although minimized by selecting a maximum 300 °C air-debinding temperature, the laboratory debinding process may inadvertently oxidize the 316L specimens.

**Figure 3:**
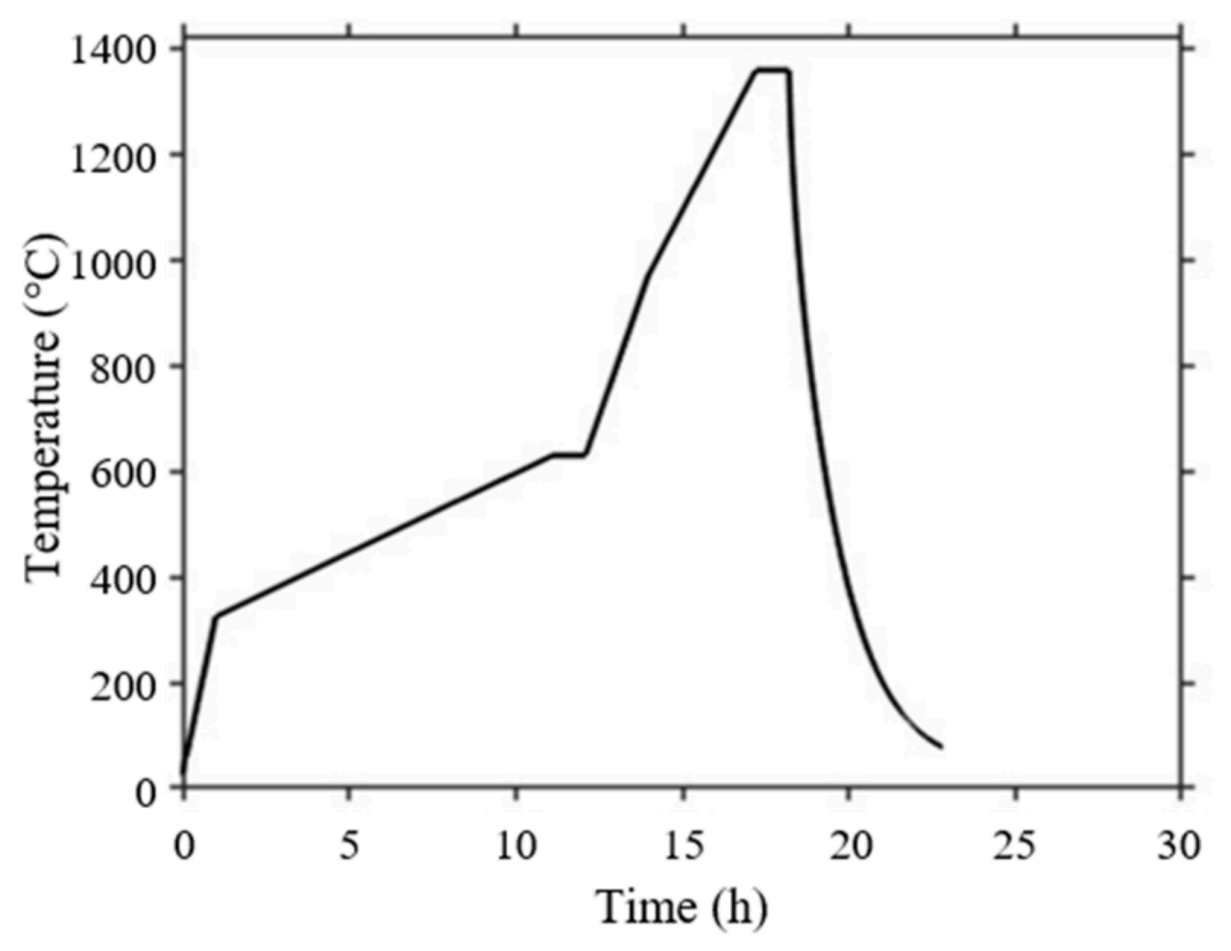
Dilatometry temperature profile used during sintering.

One specimen of each orientation was sintered in a hydrogen environment within a dilatometer (HJE Company, Inc., Queensbury, NY, USA). The sintering cycle heated the specimen from room temperature to 325 °C at 5 °C/min, heated from 325 °C to 630 °C at 0.5 °C/min, held at 630 °C for 1 h, heated from 630 °C to 970 °C at 3 °C/min, heated from 970 °C to 1360 °C at 2.0 °C/min, and held at 1360 °C for 1 h. After the 1-h hold, the dilatometry heating elements were de-energized, and the specimen was allowed to cool to room temperature at an uncontrolled rate. The sintering temperature profile as measured on the dilatometer is shown in Figure 3.

**Figure 4:**
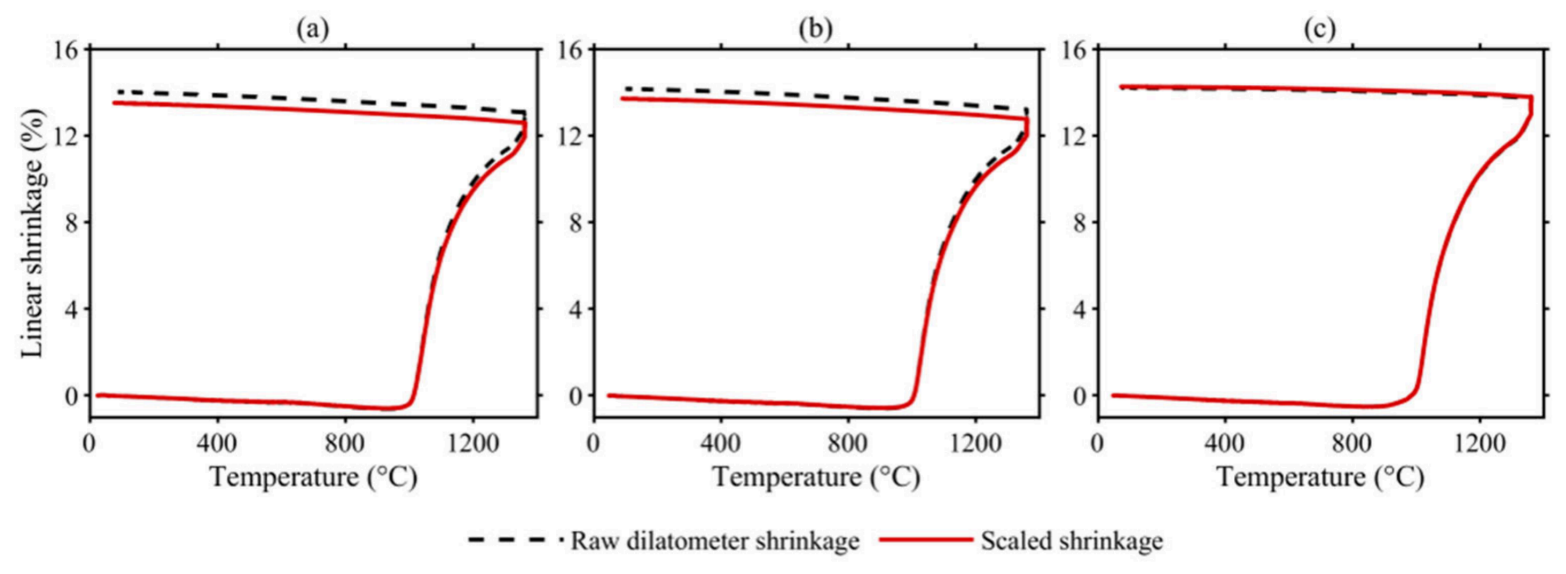
Raw and scaled dilatometer shrinkage curves for (**a**) *x*-, (**b**) *y*-, and (**c**) *z*-oriented specimens.

Dilatometer temperature and displacement data were recorded every 5 s during each sintering run. The overall specimen length was measured before and after sintering to determine the total dimensional change. Because the raw displacement signal contained instrument drift, displacement data were corrected using a constant scaling factor so that the final dilatometer-derived shrinkage matched the directly measured length change. A multiplicative correction was used because the raw and scaled curves showed the same overall shrinkage trend, while the primary discrepancy was the final displacement magnitude. The scaling factors were 0.96347, 0.9677, and 1.0040 for the *x*-, *y*-, and *z*-oriented specimens. The corresponding raw and scaled shrinkage curves are shown in Figure 4.

Scaled displacement data were normalized to the initial length $L_{0}$ and reported as temperature- and time-dependent linear shrinkage:

(1) $$S=\frac{L_{0}-L}{L_{0}}\times 100\%$$

where L is the instantaneous specimen length. Negative values of S indicate lengthening, whereas positive values correspond to shortening. After sintering, bulk density was determined by Archimedes’ principle

(2) $$\rho_{\mathrm{bulk}}=\rho_{\mathrm{water}}\frac{m_{\mathrm{air}}}{m_{\mathrm{air}}-m_{\mathrm{wet}}}$$

where $m_{\mathrm{air}}$ is the specimen mass in air, $m_{\mathrm{wet}}$ is the specimen mass measured while immersed in deionized water having temperature-dependent density $\rho_{\mathrm{water}}$.

Helium gas pycnometer (Anton Paar, Ashland, VA, USA, software v1.003.005) was utilized to measure skeletal density at 25.0 °C on seven 20-mm-long sections cut from a single BMD 316L rod of feedstock material. The seven sections were combined into three samples, with two sections in the first two samples, and three sections in the third sample. Samples were dried, cooled to room temperature, and weighed, then measured in a small cell using monolith flow mode, sample-first flow direction, pressure equilibration, 19 psi target pressure, and an end-mode criterion of better than 0.05%. The instrument automatically repeatedly runs until five consecutive measurements met the ≤0.05% variance threshold. The reported density for each specimen was the mean of those final five runs. Measured densities were 5.4766, 5.6219, and 5.4510 g/cm^3^, respectively. Assuming the feedstock material was a two-phase composite consisting of 316L stainless steel powder and a wax-polypropylene binder having densities of 7.95 and 0.94 g/cm^3^, respectively, the volume fraction of 316L stainless steel may be calculated as $V_{316L}=\frac{\rho_{pyc}-\rho_{binder}}{\rho_{316L}-\rho_{binder}}$, where $\rho_{pyc}$ is the measured density of the two-phase composite, $\rho_{binder}$ is the assumed 0.94 g/cm^3^ density of the binder, and $\rho_{316L}$ is the theoretical density of the stainless steel powder, taken as 7.95 g/cm^3^. The calculated $V_{316L}$ values are 0.647, 0.667, and 0.643, respectively.

### 2.3. Numerical Modeling

**Figure 5:**
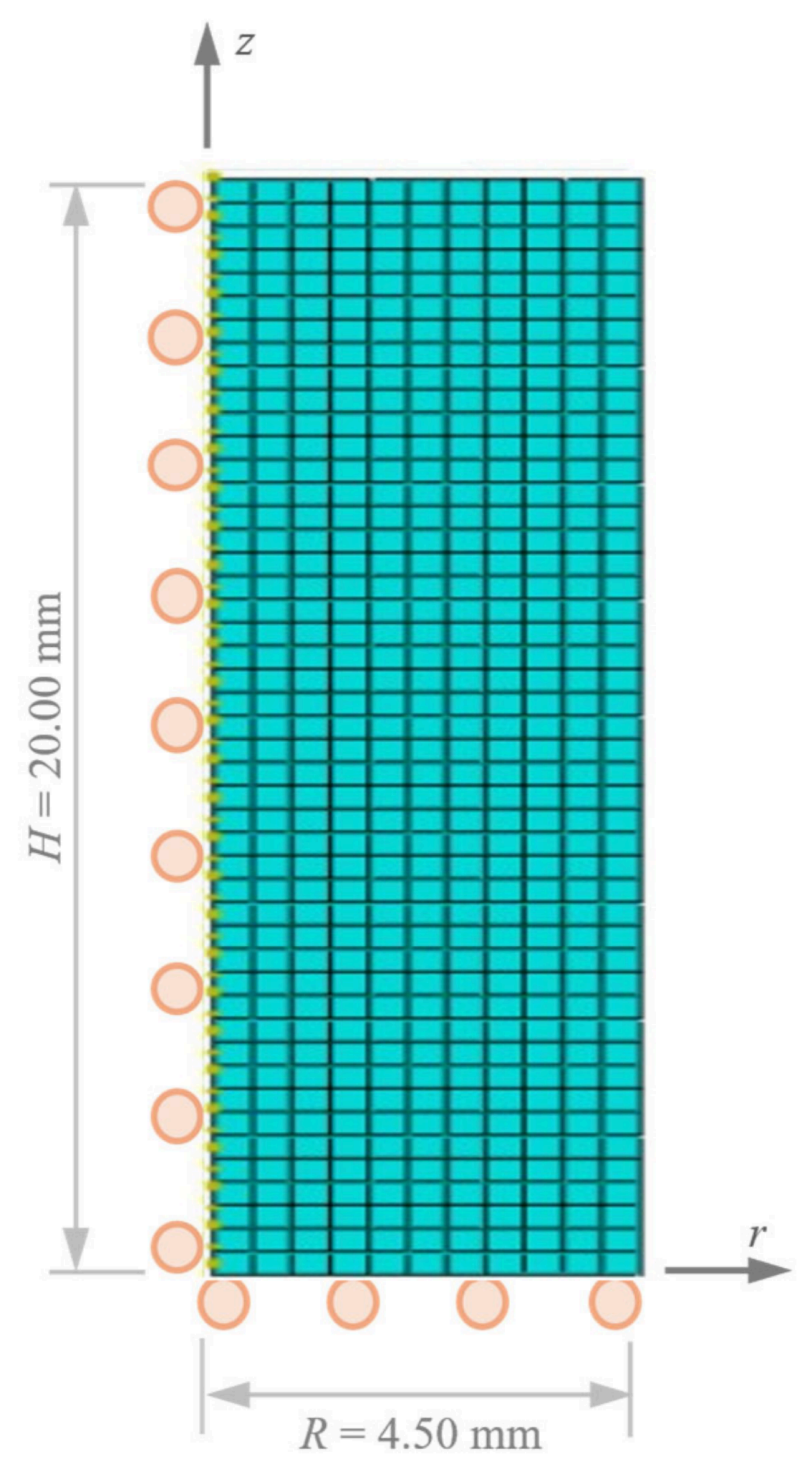
Axisymmetric finite element model of the cylindrical specimen printed in the z-orientation.

An axisymmetric finite element model of the cylindrical dilatometry sample was developed using Abaqus/Standard 2023 (Dassault Systèmes, Vélizy-Villacoublay, France). As shown in Figure 5, the axisymmetric model uses bilinear four-node CAX4T elements and roller boundary conditions, with zero displacement in the *z*-direction at *z* = 0 mm and zero displacement in the *r*-direction at *r* = 0 mm. Due to relatively slow heating rates and small sample dimensions, the experimentally measured temperature profile (cf. Figure 3) was imposed directly at each node rather than calculating nodal temperatures through a heat transfer analysis. Although tractions applied by the dilatometer’s spring-driven plunger and gravitational effects were neglected, an acceleration of 0.01 m/s^2^ in the negative *z*-direction, shown in Figure 5 was included to improve numerical stability.

Each finite element simulation was performed using a coupled temperature–displacement step. The total simulated times were 80,810 s (22.45 h) and 82,120 s (22.81 h) for *y*-and *z*-oriented specimens, respectively. Simulation time increments were bounded to 0 < Δ*t* ≤ Δ*t_max_* = 30 s; the temperature change within an increment was bounded as 0 < Δ*T* ≤ Δ*T_max_* = 30 °C.

Sintering was simulated via a rate-dependent viscous plasticity (aka creep) constitutive model incorporating isotropic thermal expansion. In strain-rate form, the constitutive equation [21] is

(3) $${\dot{\varepsilon }}_{ij}^{in}=\frac{\sigma_{ij}^{\prime }}{2\eta_{s}}+\frac{\left(\sigma_{m}-\sigma_{s}\right)}{3\eta_{b}}\delta_{ij}$$

where ${\dot{\varepsilon }}_{ij}^{in}$ is the inelastic (i.e., viscous sintering) contribution to the total strain rate; $\sigma_{ij}^{\prime }$ is the deviatoric Cauchy stress tensor; $\eta_{s}$ is the effective shear viscosity equal to $\eta_{s}=\eta_{0}\varphi$, where $\eta_{0}$ is the shear viscosity of the fully dense skeleton phase at temperature *T*, and φ is the normalized shear viscosity; $\sigma_{m}$ is the hydrostatic stress defined as $\sigma_{m}=\frac{\sigma_{kk}}{3}$ where $\sigma_{kk}$ is the trace of the Cauchy stress tensor; $\sigma_{s}$ is the sintering stress; $\eta_{b}$ is the effective bulk viscosity equal to $\eta_{b}=2\eta_{0}\psi$, where $\eta_{0}$ was previously defined and ψ is the normalized bulk viscosity; and $\delta_{ij}\;$ is the Kronecker delta operator equal to 0 if i≠j and 1 if i=j. In the assumed linear-viscous framework, $\eta_{s}$, $\eta_{b},$ and $\sigma_{s}$ evolve with temperature T, relative density ρ, and grain size *G*. This motivates the incorporation of thermally activated viscosity laws and grain growth kinetics, which connect microstructural mechanisms to the constitutive response.

The temperature-dependent shear viscosity of the fully dense matrix $\eta_{0}$ during the sintering of dual-phase austenite–delta-ferrite stainless steel was modeled using an equation inspired by Reuss [32] and the rule of mixtures [33], as proposed by Cabo Rios et al. [34]. This model combines the shear viscosities of the austenite γ and delta-ferrite δ phases, weighted by their respective phase fractions, which evolve during sintering at high temperatures. The equation is expressed as:

(4) $$\eta_{0}=\frac{1}{\frac{f_{\gamma }}{\eta_{\gamma }}+\frac{f_{\delta }}{\eta_{\delta }}}=\frac{1}{\frac{f_{\gamma }}{A_{\gamma }T\;exp\left(\frac{Q_{\gamma }}{RT}\right)}+\frac{f_{\delta }}{A_{\delta }T\;exp\left(\frac{Q_{\delta }}{RT}\right)}}$$

where $\eta_{0}$ is the shear viscosity of the fully dense matrix in Pa·s; $f_{\gamma }$ and $f_{\delta \;}$ are the dimensionless phase fractions of austenite and δ-ferrite, respectively; $A_{\gamma }$ and $A_{\delta }\;$ are the phase-specific pre-exponential factors in Pa·s·K^−1^; T is the absolute temperature in K; $Q_{\gamma }$ and $Q_{\delta }$ are the activation energies in J/mol; and R is the universal gas constant equal to 8.314 $\frac{J}{mol\cdot K}$. The equation models the temperature-dependent shear viscosity during sintering of 316L stainless steel, reflecting the dynamic changes in phase fractions as the material transitions from austenite to a dual-phase microstructure at high temperatures. Following the Reuss model [32], the equation assumes equal stresses (iso-stress) in the austenite and delta-ferrite phases, with strain rates inversely proportional to each phase viscosity. This simplification allows for the calculation of shear viscosity by assuming uniform stress distribution across phases, but with different strain responses. The equation is further refined by incorporating the rule of mixtures [33], which considers the individual shear viscosities of both phases and their evolving phase fractions with temperature and time.

The effective shear viscosity is [21]

(5) $$\eta_{s}={\eta_{0}\left(1-\theta \right)}^{2}$$

where $\eta_{0}$ is defined in Equation (4); θ is porosity, defined as θ=1−ρ with 0 ≤ θ ≤ 1; and ρ is relative density. The effective bulk viscosity is formulated in accordance with a power-law form [29]

(6) $$\eta_{b}=\frac{4{\eta_{0}\left(1-\theta \right)}^{p}}{{3\theta }^{\lambda }}$$

where $\eta_{0}$ and θ were previously defined, and p and λ are empirically determined exponents. The original Olevsky model [21] is recovered as the special case p=3 and λ=1.

The sintering stress, or effective Laplace pressure, originates from capillary forces associated with surface curvature and acts as the internal driving force for pore size reduction and densification via Olevsky model [21].

(7) $$\sigma_{s}=\frac{3\gamma_{s}}{r}{\left(1-\theta \right)}^{2}$$

where $\gamma_{s}$ is the surface energy in J/m^2^; r is the mean particle radius in m; and θ was previously defined. For crystalline systems, $r=\frac{G}{2}$, where G is the current average grain size, thereby linking macroscopic driving stress directly to microstructural evolution [21].

The transition temperature $T_{trans}$ is a critical temperature during sintering. For T < $T_{trans}$, the material predominantly experiences thermal expansion. Within the constitutive model, the capillary-driven sintering contribution was numerically deactivated for T < $T_{trans}$ where the measured dimensional response was dominated by thermal expansion. For T ≥ $T_{trans}$, sintering and densification are dominant. Within the constitutive model, the sintering response was activated for T ≥ $T_{trans}$ via nonzero surface energy, i.e., $\gamma_{s}\neq$ 0. Hence, $T_{trans}$ is an empirical activation function in the model and does not physically represent the surface energy behavior of real materials.

Thermal strains due to temperature changes are expressed as

(8) $${\dot{\varepsilon }}_{ij}^{th}=\alpha ∆\dot{T}\delta_{ij}$$

where α is the effective isotropic coefficient of thermal expansion; $∆\dot{T}$ is the rate of temperature change; and $\delta_{ij}$ was previously defined.

Grain growth was modeled using the following relation [35]

(9) dGdt=K03G2(1−ρC2−ρC−ρ)32exp(−QGRT)

where $K_{0}$ is a material constant in µm^3^/s, $\rho_{C}$ is the dimensionless critical relative density where pores stop strongly resisting grain-boundary motion, $Q_{G}$ is the activation energy for grain growth in J/mol, and *ρ*, *T*, and *R* were previously defined.

At high sintering temperatures, SS 316L can transition from a predominantly *γ*-austenitic microstructure to a dual-phase state composed of *γ*-austenite and *δ*-ferrite [34]. Within the solid *γ* + *δ* region, the corresponding phase fractions evolve with temperature and satisfy $f_{\gamma }(T)+f_{\delta }(T)=1$. Because the constitutive model incorporates phase-dependent viscosity, the temperature-dependent phase fractions were included to account for the evolving *γ*–*δ* phase balance during sintering.

**Figure 6:**
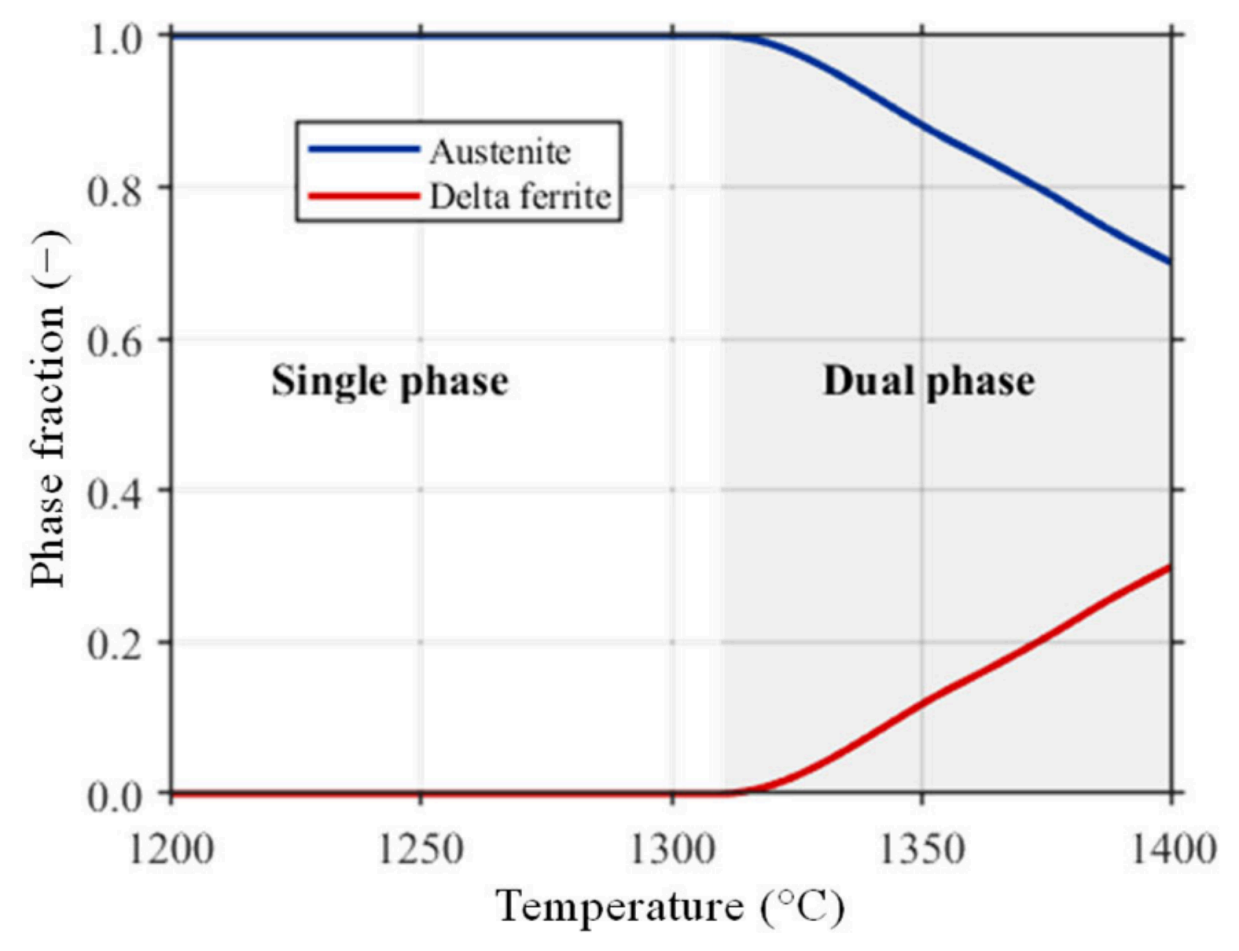
Reconstructed phase equilibrium fractions of *γ*-austenite and *δ*-ferrite in SS 316L as a function of temperature (adapted from [34]).

The chemical composition of Desktop Metal BMD 316L v.2 (cf. Table 1) [36] is consistent with the composition of SS 316L in Cabo Rios et al. [34]. Here, the JMatPro-calculated phase-fraction data of Cabo Rios et al. [34] are utilized to define temperature-dependent γ-austenite and *δ*-ferrite fractions in the present model. As shown in Figure 6, the material remains fully *γ*-austenitic below approximately 1310 °C, after which *δ*-ferrite progressively develops. Because the maximum temperature of the present thermal cycle was 1360 °C, the material remained within the solid *γ* + *δ* region. The corresponding *γ*-austenite and *δ*-ferrite fractions were defined such that $f_{\gamma }(T)+f_{\delta }(T)=1$. These phase fractions were incorporated into the constitutive model to continuously interpolate between the phase-specific viscosities as the *γ*–*δ* balance changes with temperature.

The isotropic constitutive model uses fourteen material parameters; typical values and data sources are summarized in Table 2. After correction and normalization of the dilatometry data, the transition temperature $T_{trans}$ was identified from the BMD SS 316L curve as the point marking the onset of significant densification. Below this temperature, dimensional change was dominated by thermal expansion, not sintering-induced densification. Using a tangent–intercept (two-slope) construction on the shrinkage-temperature trace, the lower and upper bounds for $T_{trans}$ were estimated to be 990–1030 °C. Bounds for p and *λ* were selected as 0.5675 ≤ p ≤ 22.70 and 0.098 ≤ λ ≤ 3.92, representing proportionally expanded ranges from baseline parameterization reported in [31] to capture the full extent of the dilatometry-measured shrinkage behavior.

The *V_316L_* = 0.647, 0.667, and 0.643 pycnometer data (cf. Section 2.2) provide a means to bound initial relative density *ρ_0_* between 64.3 and 66.7%. Alternately, the measured *x*-, *y*-, and *z*-oriented linear shrinkages and experimental densities (cf. Section 3) provide another means to bound *ρ_0_* by calculating the initial green state density *ρ_gs_* = *ρ_f_* (1 − *S_x_*)(1 − *S_y_*)(1 − *S_z_*), where *ρ_f_* is the final density; and *S_x_*, *S_y_*, and *S_z_* are shrinkages in the *x*-, *y*-, and *z*-directions, respectively [37]. Assuming *ρ_f_* = 0.967 and *S_x_* = 0.135, *S_y_* = 0.137, and *S_z_* = 0.143, the *ρ_gs_* = (0.967)(1 − 0.135)(1 − 0.137)(1 − 0.143) = 0.618, or 61.8%. Combining these two bounds results in *ρ_0_* bounded to 61.7–66.7%.

**Table 2:** Material parameters, nominal values or bounds, and data sources used in the constitutive model. Nominal values are given as a single value; bounds are given as a range.

Type	Parameters	Values	Ref.
Design	Surface energy, $\gamma_{s}$	1–2 J/m^2^	[11,12,38]
	Grain growth critical density, $\rho_{C}$	0.92–0.96	[31,35,39]
	Grain growth constant, $K_{0}$	$2.965\times 10^{-4}-6.75\times 10^{5}$ μm^3^/s	[38,40]
	Pre-exponential factor, $A_{\delta }$	1 × 10^−18^–1 × 10^−17^ Pa·s·K ^−1^	[34]
	Pre-exponential factor, $A_{\gamma }$	1–3 Pa·s·K ^−1^	[34]
	Activation energy for ferrite, $Q_{\delta }$	700 × 10^3^–800 × 10^3^ J/mol	[34,41]
	Activation energy for austenite, $Q_{\gamma }$	100 × 10^3^–250 × 10^3^ J/mol	[34,41]
	Transition temperature, $T_{trans}$	990–1030 °C	This work
	Fitting parameter, *p*	0.5675–22.70	[31]
	Fitting parameter, λ	0.098–3.92	[31]
	Initial relative density, $\rho_{0}$	61.7–66.7%	This work
Fixed	Coefficient of thermal expansion, α	6.9 × $10^{-6}$ K^−1^	Measured
	Initial average grain diameter, $G_{0}$	10 µm	[7]
	Grain growth activation energy, $Q_{G}$	$\{\begin{array}{c} \;\;50.0\times 10^{3}\;J/mol\;\forall \;T<1200\;{}^{\circ}C\\ 315.8\times 10^{3}\;J/mol\;\forall \;T\;\geq \;1200\;{}^{\circ}C \end{array}$	[42]

### 2.4. Numerical Convergence Study

Numerical convergence was evaluated based on both temporal and spatial convergence. For temporal convergence, a discretization sensitivity study was conducted using maximum time steps Δ*t_max_* = 5, 30, 60, 100, 300, and 600 s. As shown by the temporal convergence data in Table 3, the final shrinkage as a function of Δ*t_max_* varied from 13.653% for Δ*t_max_* = 30 s to 13.755% for Δ*t_max_* = 600s, corresponding to a range of 0.102 percentage points based on the rounded values reported in the table. The difference in final shrinkage, Δ*S_f_*, shown in the third column was calculated relative to the refined Δ*t_max_* = 5 s case. The remaining columns report the numerically calculated final relative density *ρ_f_*, the difference in the final relative density Δ*ρ_f,_* and the solver wall-clock time. Relative to the refined Δ*t_max_* = 5 s case, the Δ*t_max_* = 30 s case resulted in a Δ*S_f_* = 0.037 percentage points and a Δ*ρ_f_* = −0.001, while reducing the solver wall-clock time from 956 s to 113 s. Therefore, Δ*t_max_* = 30 s was selected for all reported simulations as a conservative practical time-step limit. Although larger time-step limits further reduced the solver wall-clock time, Δ*t_max_* = 30 s was retained because it provides finer temporal resolution during the nonlinear sintering process and avoids relying only on agreement in final scalar values.

**Figure 7:**
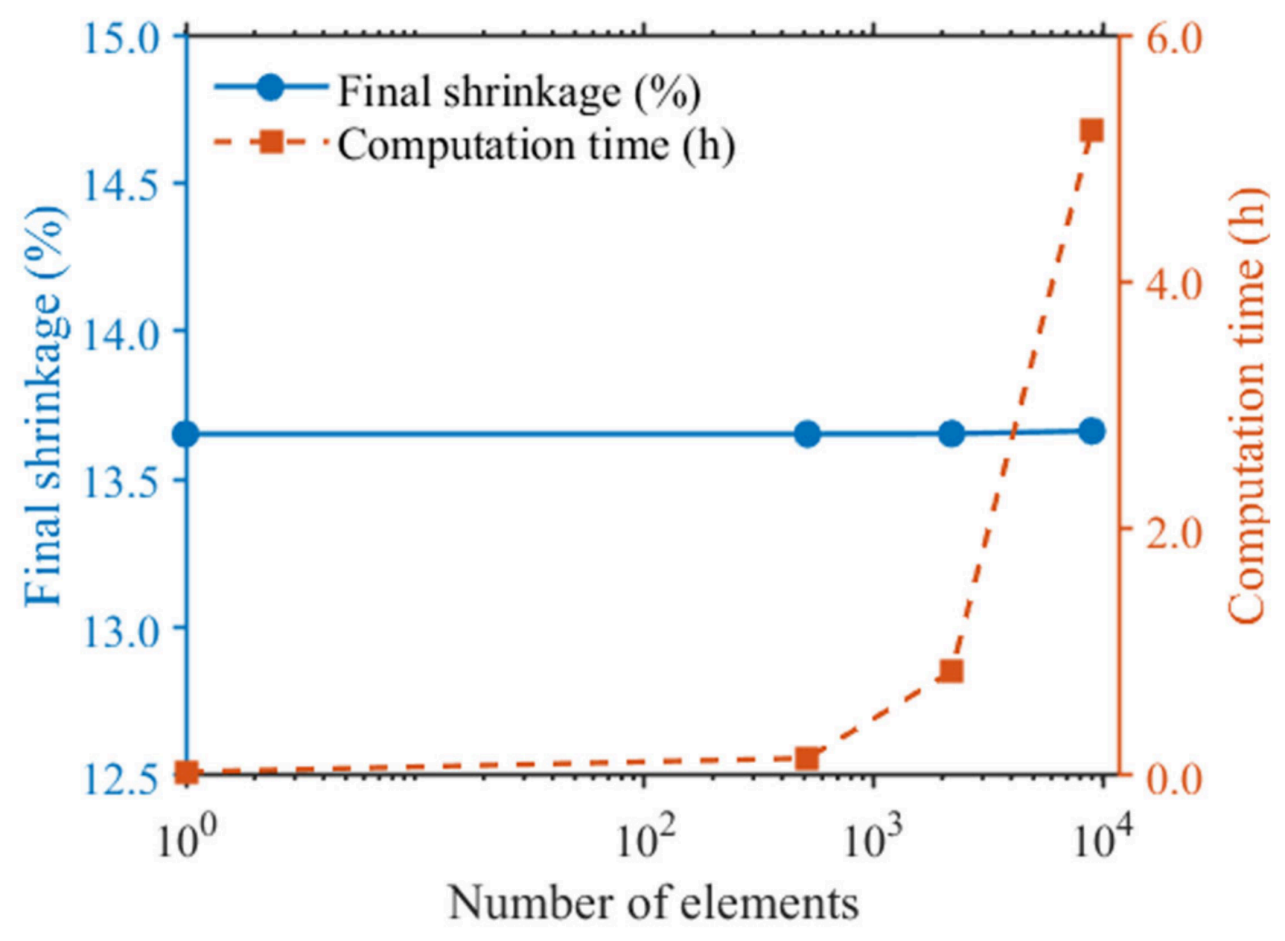
Mesh convergence results showing final shrinkage and wall-clock computation time as a function of the number of elements.

Spatial convergence was evaluated by progressively refining the finite element mesh from 1 to 9000 elements. The computed final shrinkage remained nearly unchanged, varying only from 13.653% to 13.663%, which corresponds to a difference of 0.010 percentage points across the tested mesh range (cf. Figure 7). This small variation indicates minimal mesh sensitivity in the final shrinkage response. However, the computation time increased substantially with mesh refinement. The 1-element mesh required approximately 0.031 h, whereas the 9000-element mesh required approximately 5.23 h. In contrast, the final relative density remained constant at approximately 0.976 for all tested mesh sizes. The computational cost increased sharply beyond the 517-element mesh, while the shrinkage and density responses showed minimal variation. Therefore, a 517-element mesh was adopted for all subsequent analyses as a balance between numerical accuracy and computational efficiency.

### 2.5. Particle Swarm Optimization (PSO)

A particle swarm optimization (PSO) [43] algorithm was utilized to calibrate the material parameters. In PSO, a population of N particles was initialized within the search domain, and the objective function value was evaluated for each particle. Prior to the next iteration, the particles updated their velocities and positions according to Ratnaweera et al. [44]

(10) $$v_{i}^{\left(t+1\right)}=wv_{i}^{\left(t\right)}+c_{1}r_{1}\left(P_{i}^{\left(t\right)}-x_{i}^{\left(t\right)}\right)+c_{2}r_{2}\left(G^{\left(t\right)}-x_{i}^{\left(t\right)}\right)$$

(11) $$x_{i}^{\left(t+1\right)}=x_{i}^{\left(t\right)}+v_{i}^{\left(t+1\right)}$$

where $v_{i}^{\left(t\right)}$ and $v_{i}^{\left(t+1\right)}$ are the velocities of the *ith* particle at iterations t and *t* + 1, respectively; w is the inertia weight parameter controlling exploration and exploitation; $c_{1}$ and $c_{2}$ are the cognitive and social acceleration coefficients respectively; $r_{1}$ and $r_{2}$ are random numbers uniformly distributed in the interval [0,1]; $P_{i}^{\left(t\right)}$ represents the personal best position found by *ith* particle up to iteration *t*; $G^{\left(t\right)}$ denotes the global best position found by the entire swarm up to iteration *t*; and $x_{i}^{\left(t\right)}$ and $x_{i}^{\left(t+1\right)}$ are the positions of the $i^{th}$ particle at iterations *t* and *t* + 1, respectively.

Instead of employing a constant inertia weight, which cannot effectively balance global exploration at the early stages with local exploitation near convergence, this study adopted the linearly varying inertia weight (LVIW) strategy proposed by Shi and Eberhart [45]. In this approach, the inertia weight w decreases linearly with iteration according to $w(t)=(w_{max}-w_{min})((t_{max}-t)/t_{max})+w_{min}$, where t is the current iteration, $t_{max}$ is the maximum number of iterations, and $w_{max}=0.9$ and $w_{min}=0.4$. This adaptive control enabled the swarm to initially maintain search diversity while promoting convergence near the optimum. Similarly, the cognitive and social acceleration coefficients $c_{1}$ and $c_{2}$, respectively, were adaptively varied using the Hierarchical PSO with Time-Varying Acceleration Coefficients (HPSO-TVAC) method [44,46], which adjusts the parameters over iterations as $c_{1}(t)=(c_{1f}-c_{1i})(t/t_{max})+c_{1i}$ and $c_{2}(t)=(c_{2f}-c_{2i})(t/t_{max})+c_{2i}$, where $c_{1i}=2.5$, $c_{1f}=0.5$, $c_{2i}=0.5$, $c_{2f}=2.5$, and *t* and *t_max_* were previously defined. This dynamic formulation promoted extensive exploration in the early search phase and stronger convergence during the later phase. To prevent divergence and maintain numerical stability, velocity clamping was employed such that $v_{i}^{\left(t+1\right)}=min(v_{i}^{\left(t+1\right)},V_{max})$, where the maximum velocity was limited by $V_{max}=0.2\left(x_{max}-x_{min}\right)$, where $x_{max}$ and $x_{min}$ are the upper and lower bounds of search space [46]. The swarm size was set to 110 particles, corresponding to 10 particles per design variable for eleven optimized parameters, ensuring sufficient search diversity. This choice follows the dimensional-scaling practice commonly adopted in high-dimensional PSO studies and is supported by Piotrowski et al. [47], who demonstrated that PSO performance typically improves with larger swarms (70–500 particles) for complex, multi-parameter problems, extending beyond the conventional 20 to 50 particle range [48]. The optimization was terminated after 10 iterations [46]. The stopping criterion was defined by a fixed computational budget of 1100 function evaluations corresponding to 10 iterations with 110 particles, ensuring balanced exploration, reproducibility, and computational efficiency throughout the optimization [46].

The PSO optimized the eleven design material parameters listed in Table 2. Initial particle placement in the first iteration was accomplished via Latin Hypercube Sampling (LHS) [49] and one manual seeding [50]. LHS ensures uniform coverage, and the manual seed embedded prior knowledge of shrinkage behavior [51]. Prior studies have shown that informed initialization enhances PSO convergence while avoiding stagnation [51,52,53], a finding consistent with the present calibration framework.

The model parameters were calibrated using particle swarm optimization (PSO) by minimizing a weighted single-objective cost function. The cost function utilized the scaled-shrinkage curve, the final scaled shrinkage, and the final relative-density via

(12) $$J\left(x\right)=\sqrt{\frac{1}{n}\sum_{i=1}^{n}{\left(S_{\left(i\right)}^{Model}\left(x\right)-S_{\left(i\right)}^{Exp}\right)}^{2}+w_{s}{\left(S_{f}^{Model}\left(x\right)-S_{f}^{Exp}\right)}^{2}+w_{\rho }{\left[100\left(\rho_{f}^{Model}\left(x\right)-\rho_{f}^{Exp}\right)\right]}^{2}}$$

where J(x) is the PSO cost function; x is the vector consisting of the eleven design parameters; *n* is the number of unique points in time for the simulation; *i* is an integer index; $S_{\left(i\right)}^{Model}\left(x\right)$ is the shrinkage calculated by the numerical model at time *t* corresponding to the *ith* point; $S_{\left(i\right)}^{Exp}$ is the experimentally measured shrinkage at time *t* corresponding to the *ith* point; $w_{s}$ is a weighting factor set to 0.25; $S_{f}^{Model}$ and $S_{f}^{Exp}$ are the final model-calculated and experimentally measured shrinkages, respectively; $w_{\rho }$ is a weighting factor set to 0.05; and $\rho_{f}^{Model}\left(x\right)$ and $\rho_{f}^{Exp}$ are the final model-calculated and experimentally measured relative densities, respectively. The factor of 100 converts the relative density differences into percentage points.

**Figure 8:**
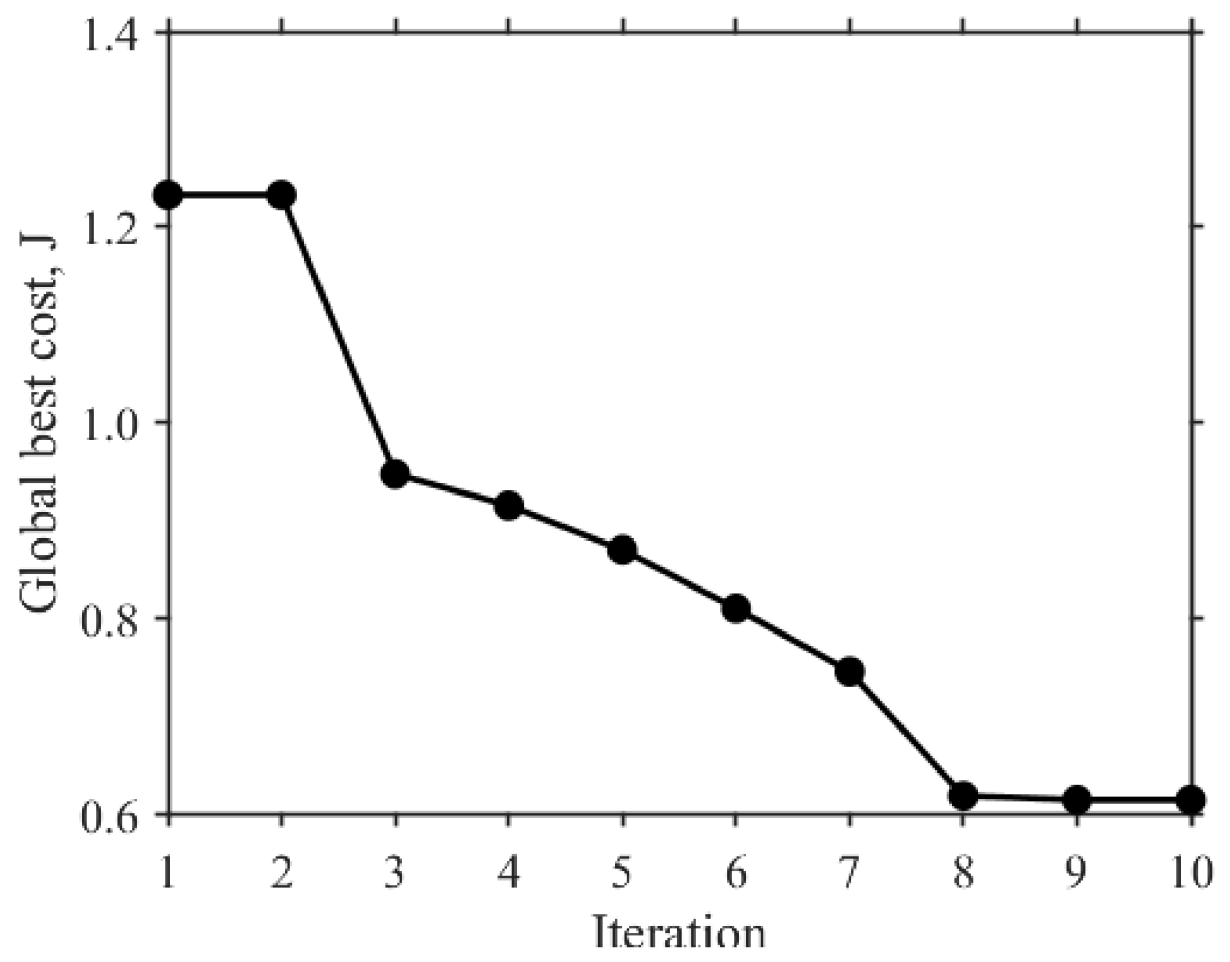
PSO Convergence history for the *y*-oriented specimen.

The convergence curve in Figure 8 illustrates the evolution of the best cost function J(x) over 10 PSO iterations. A sharp decrease in the first few iterations indicates effective exploration of the parameter space and early identification of promising regions. The cost function decreased from 1.2324 in the initial iteration to 0.7451 in iteration 7 and 0.6138 in iterations 8 through 10.

## 3. Results

**Figure 9:**
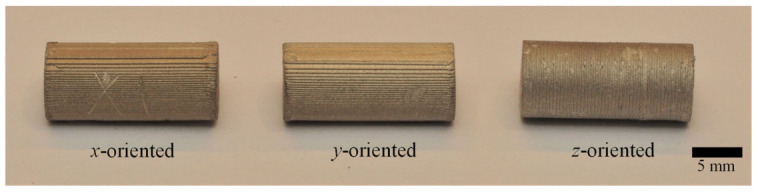
As-sintered specimens for *x*-, *y*-, and *z*-oriented specimens.

The as-sintered cylindrical specimens printed in *x*-, *y*-, and *z*-orientations are shown in Figure 9. Because the *x*- and *y*-orientated specimens were orientated in the build plane, the layer lines are longitudinal. In contrast, the z-orientated specimen was printed in the build direction; thus the layer lines are circumferential.

**Figure 10:**
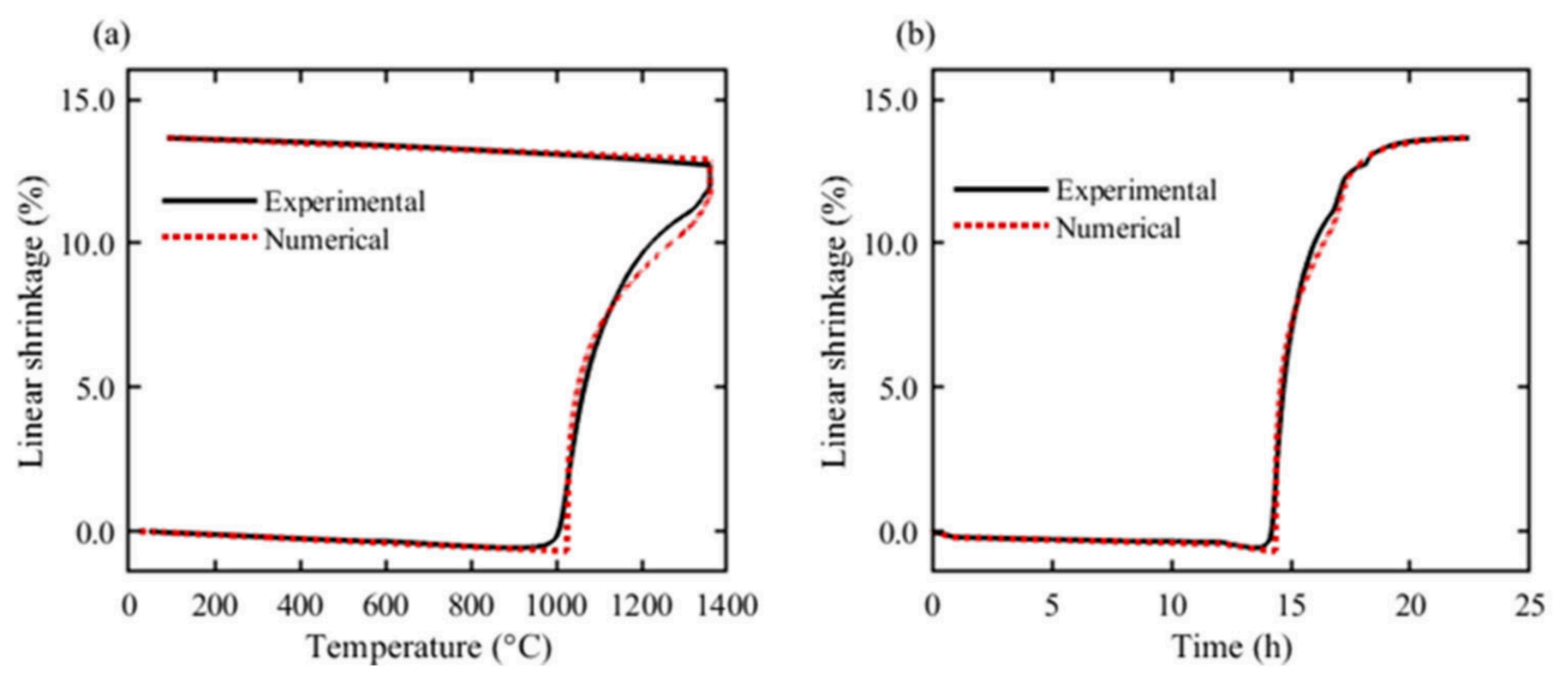
Experimental and numerical linear shrinkage curves for *y*-oriented specimen showing (**a**) shrinkage versus temperature, and (**b**) shrinkage versus time.

**Figure 11:**
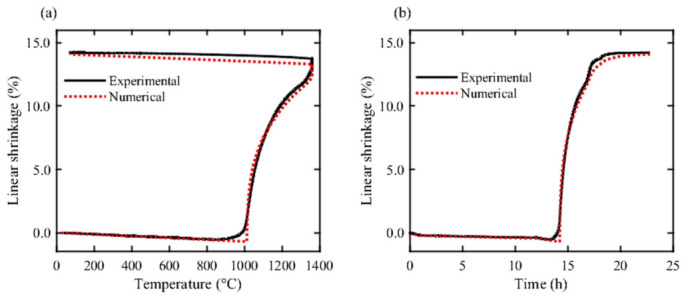
Experimental and numerical linear shrinkage curves for *z*-oriented specimen showing (**a**) shrinkage versus temperature, and (**b**) shrinkage versus time.

**Figure 12:**
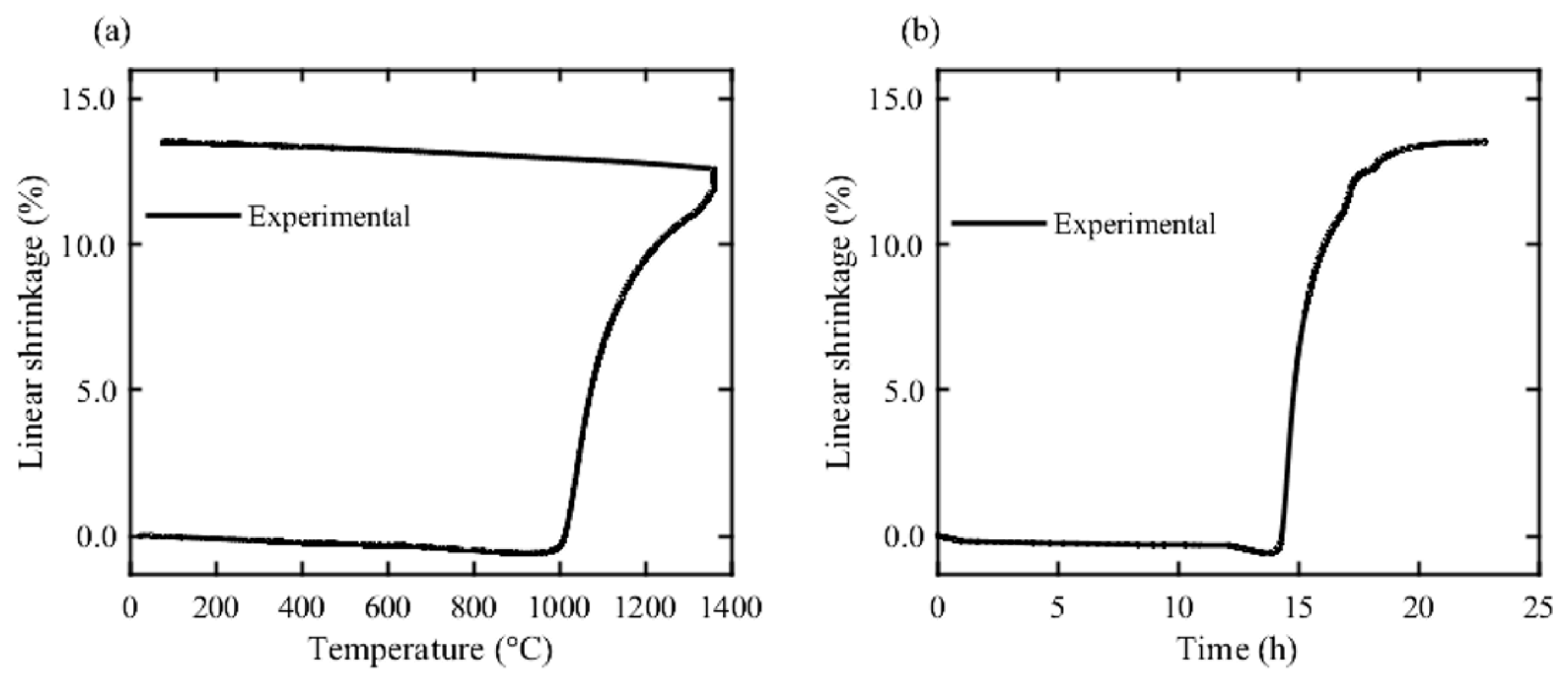
Experimental linear shrinkage curves for *x*-oriented specimen showing (**a**) shrinkage versus temperature, and (**b**) shrinkage versus time.

Linear shrinkage data from dilatometer measurements and calibrated numerical simulations are compared in Figure 10 for the *y*-oriented specimen and Figure 11 for the *z*- oriented specimen. In both orientations, the calibrated simulations reproduce the experimentally measured shrinkage evolution as functions of both time and temperature. The curves initially exhibit thermal expansion, followed by the onset of densification at elevated temperatures as sintering shrinkage becomes dominant. Rapid shrinkage then occurs during the high-temperature sintering stage, after which the shrinkage rate gradually decreases during the peak-temperature hold. Because orientation-specific parameter calibration was used, these results should be interpreted as calibrated agreement rather than independent cross-orientation prediction. Linear shrinkage data from dilatometer measurements for the *x*-oriented specimen are shown in Figure 12.

The final experimental linear shrinkage values were 13.7% for the *y*-oriented specimen and 14.3% for the *z*-oriented specimen. Both values were lower in magnitude than the typical BMD shrinkage range of 14.9% to 15.1% [54]. The calibrated numerical simulations gave final shrinkage values of 13.7% for the *y*-oriented specimen and 14.1% for the *z*-oriented specimen, with absolute shrinkage differences of 0.0 and 0.2 percentage points relative to the experimental measurements, respectively. The *y*- and *z*-orientated experimental final relative densities were 0.976 and 0.967; simulated final relative densities were 0.975 and 0.974 for the *y*- and *z*-oriented specimens, respectively. Hence, the absolute density differences between experimental and numerical were 0.001 and 0.007 for *y*- and *z*-orientated specimens, respectively. The *x*-oriented specimen showed a final experimental shrinkage of 13.5%, which was close to that of the *y*-oriented specimen; therefore, the *y*-oriented specimen was considered representative of the in-plane *x*- and *y*-oriented specimens. Table 4 summarizes the dimensional measurements, with final experimental linear shrinkage values ranging from 13.5% to 14.3%. Quantitatively, the *z*-oriented specimen shrank 0.6 percentage points more than the *y*-oriented specimen and 0.8 percentage points more than the *x*-oriented specimen.

As all dilatometry specimens were sintered horizontally, the gravitational loading condition was similar for all three orientations. Therefore, the observed orientation-dependent shrinkage was unlikely to result only from the sintering configuration. Instead, it suggests that printing-induced microstructural features, such as interlayer interfaces, anisotropic pore distributions, or local density variations introduced during fabrication, may have contributed to the measured shrinkage response. This interpretation is consistent with the layer-by-layer nature of the BMD process, although further microstructural characterization would be needed to isolate the individual contributions of these features.

As shown in Table 5, the final measured densities ranged from 7.69 to 7.76 g/cm^3^, corresponding to 96.7 to 97.6%, respectively, of the theoretical density of 7.95 g/cm^3^ [55]. These values indicate that near-complete densification was achieved under the applied processing conditions. The difference between the lowest and highest measured relative densities was 0.9 percentage points.

The optimized parameters determined by PSO are shown in Table 6. Calibrated values remained within the prescribed bounds listed in Table 2. The calibrated transition temperatures were 1025 °C and 1014 °C for the *y*- and *z*-oriented specimens, respectively, consistent with the onset of rapid shrinkage in the dilatometry curves. The corresponding grain growth critical densities were 0.96 for both orientations, which fall within the expected range of 0.92–0.96 [31,35,39]. Overall, the PSO calibration produced reasonable parameter sets and close agreement with the measured shrinkage behavior. Each calibration required approximately nine days of wall-clock time using 8 CPU cores per specimen.

## 4. Discussion

The dilatometry and calibrated simulation curves display an initial thermal-expansion-dominated response, followed by the onset of densification as sintering shrinkage becomes dominant at elevated temperature. The shrinkage rate then gradually decreases during the high temperature hold as the specimens approach their final densified state. The close agreement between the measured and simulated curves indicates that the calibrated model captures the shrinkage responses of the as-tested specimens.

**Figure 13:**
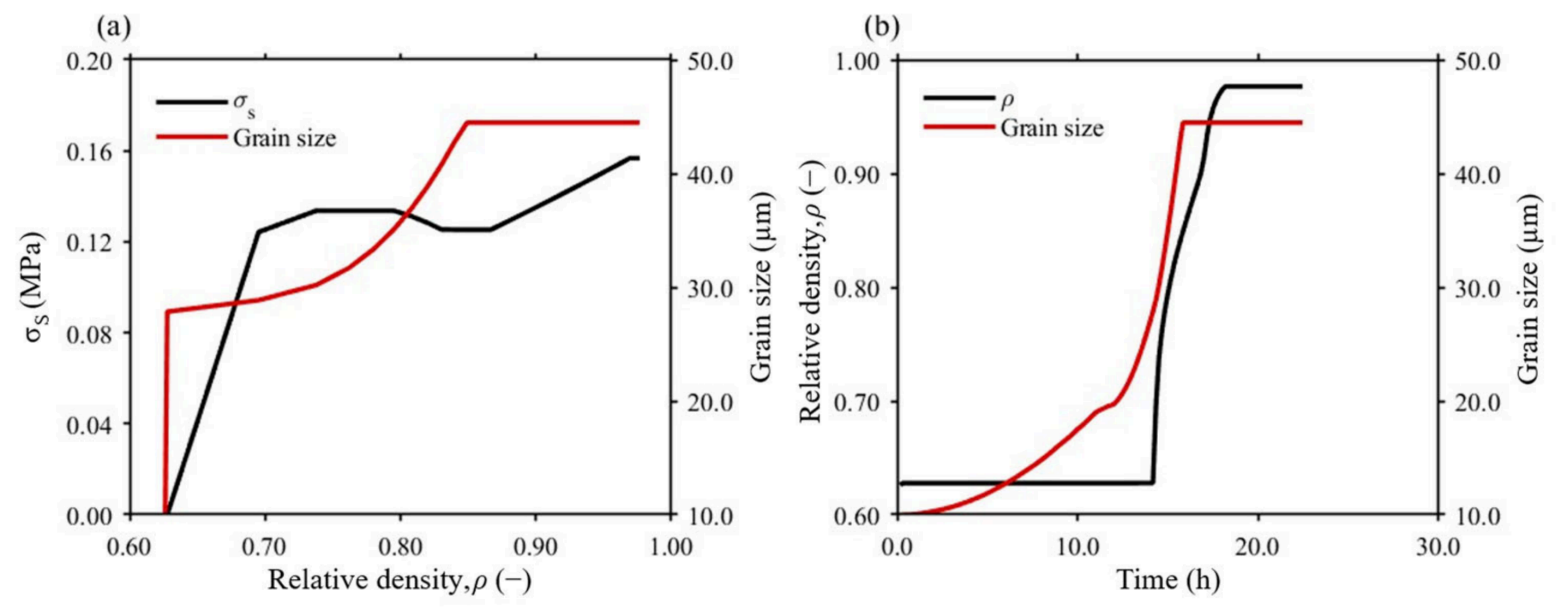
Simulated evolution of (**a**) sintering stress and grain size as functions of relative density, (**b**) relative density and grain size as functions of time.

Beyond the displacement response, the evolution of the model-calculated internal state variables provides additional insight into the simulated sintering behavior. For example, Figure 13a shows the coupled evolution of sintering stress and grain size as functions of relative density for the *y*-orientated simulation. The model assigned grain size starts from the prescribed initial value of 10 μm and increases during densification, approaching approximately 45 μm near the final stage. In contrast, the sintering stress evolves non-monotonically over the density range. It increases rapidly during the early densification stage, remains close to 0.13 MPa over the intermediate density range, decreases slightly as grain growth continues, and then increases again near the final stage to approximately 0.16 MPa. This behavior suggests that grain growth and sintering stress are coupled but do not evolve proportionally. As grain coarsening progresses, changes in effective curvature and pore/grain geometry may modify the capillary driving force, producing variations in sintering stress during densification.

Figure 13b shows the evolution of relative density and grain size as functions of time. During the initial heating stage, the relative density remains nearly constant at approximately 0.63, while gradual grain growth begins from the prescribed initial grain size of 10 μm. As the simulated specimen enters the high-temperature sintering regime, both grain growth and densification accelerate. The relative density increases rapidly between approximately 14 and 18 h, reaching its final value of 0.975, while the grain size approaches approximately 45 μm. After this rapid densification stage, both relative density and grain size show little additional change and approach stable values near the end of the thermal cycle. The earlier onset of grain growth compared with rapid densification suggests that microstructural coarsening begins before substantial pore elimination occurs.

**Figure 14:**
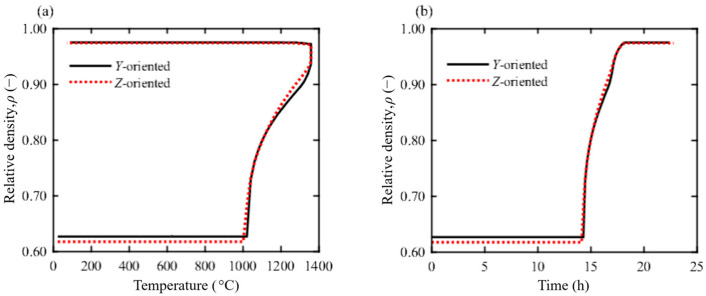
Simulated relative density evolution as a function of (**a**) temperature and (**b**) time.

The corresponding evolution of relative density as functions of temperature and time for the *y*- and *z*-oriented specimens are shown in Figure 14. Both orientations exhibit a low-density plateau during the early heating stage, followed by rapid densification at elevated temperature and stabilization near the final relative density. This trend is consistent with the shrinkage response observed in the dilatometry curves and confirms that the calibrated model captures the main transition from thermal expansion to sintering-driven densification. The similarity between the *y*- and *z*-oriented density curves indicates that the calibrated parameters reproduce the overall densification trend for both orientations, while the small differences between the curves reflect orientation-dependent differences in the calibrated response.

Although the differences in shrinkage among the three orientations were relatively small, the results indicate that specimen orientation influenced the dimensional response during sintering. Because all dilatometry specimens were sintered under the same thermal cycle and in the same horizontal configuration, the observed differences are unlikely to arise solely from the sintering conditions. Instead, they suggest that subtle microstructural differences associated with the printing process may contribute to the orientation dependence of shrinkage. At the same time, the small variation in final relative density suggests that the orientation effect was more evident in the measured dimensional change than in the final densification level.

Taken together, the results link the measured shrinkage response with the simulated evolution of relative density, grain size, and sintering stress. This connection provides insight into the interaction between thermal softening, densification, and grain coarsening during sintering. The calibrated framework can therefore support future process optimization and shrinkage compensation in extrusion-based metal additive manufacturing while emphasizing the importance of accounting for print orientation in dimensional control.

The scope of this study is defined by the available experimental data and the assumptions used in the numerical model. The calibration was based on a limited number of dilatometry specimens and was conducted using one feedstock system, one thermal sintering schedule, and one furnace setup. Hence, the model is descriptive rather than predictive. Additional testing across multiple feedstock batches, printing conditions, machines, and sintering furnaces would help evaluate process-to-process variability. The *y*- and *z*-oriented simulations also used orientation-specific calibrated parameter sets; therefore, the results represent calibrated descriptions of the measured responses rather than independent cross-orientation predictions. The laboratory debind–sinter procedure used for dilatometry was also decoupled from the full commercial BMD furnace cycle, which may influence early-stage sintering and shrinkage kinetics. In the numerical model, the printed structure was treated as a homogenized continuum; therefore, local inter-raster voids, binder-related heterogeneity, particle rearrangement, and spatial variations in pore morphology were not explicitly resolved. In addition, grain size and sintering stress were model-calculated internal variables and were not directly validated using post-sintering microstructural measurements. Nevertheless, the calibrated framework connects the measured shrinkage response with the simulated evolution of density, grain growth, and sintering stress.

## 5. Conclusions

The main findings of this study are summarized as follows:

1. Dilatometry measurements of SS 316L BMD specimens printed in different orientations showed an orientation-dependent shrinkage trend. The final linear shrinkage values were 13.5%, 13.7%, and 14.3% for the *x*-, *y*-, and *z*-oriented specimens, respectively, suggesting a tendency for increased shrinkage along the build direction.
2. A physics-based constitutive model incorporating density-dependent viscosities, grain-growth kinetics, and capillary-driven sintering stress was calibrated using experimental dilatometry data. The calibrated model reproduced the measured shrinkage evolution and final relative density of the tested specimens.
3. The simulated internal state variables showed coupled evolution of relative density, grain size, and sintering stress. These trends provide insights into the interaction between thermal softening, densification, grain coarsening, and the increasing resistance to further shrinkage near the final stage.
4. The final measured densities ranged from 96.7% to 97.6% of theoretical density, indicating near-complete densification under the applied processing conditions. The small spread in final relative density suggests that orientation had only a limited effect on the final densification level.

Future work will extend the proposed framework to complex geometries, where non-uniform shrinkage and orientation-dependent effects may become more pronounced. Additional repeated dilatometry testing, uncertainty analysis, and microstructural characterization will further support the interpretation of orientation-dependent shrinkage behavior. Addressing these challenges will support the development of improved shrinkage compensation strategies for components with intricate geometries, thereby enhancing the industrial applicability of BMD.

## Figures and Tables

**Table 1:** Composition of Desktop Metal BMD 316L v.2 material (wt.%).

Element	Composition (wt.%)
Ni	10–14
Cr	16–18
Mo	2–3
Mn	≤2.0
Si	≤1.0
C	≤0.03
Fe	Balance

**Table 3:** Results of temporal sensitivity study.

Maximum Time Step, Δ*t_max_* (s)	Final Shrinkage, *S_f_* (%)	Diff in *S_f_*, Δ*S_f_* (Percentage Points)	Final Density, *ρ_f_* (--)	Diff in *ρ_f_*, Δ*ρ_f_* (--)	Wall-Clock Time (s)
5	13.690	0.000	0.977	0.0000	956
30	13.653	0.037	0.976	−0.001	113
60	13.657	0.033	0.976	−0.001	49
100	13.666	0.024	0.976	−0.001	26
300	13.698	−0.008	0.978	0.001	7
600	13.755	−0.065	0.979	0.002	5

**Table 4:** Summary of experimental lengths and masses for *x*-, *y*-, and *z*-oriented dilatometry specimens.

Specimen	Length Initial (mm)	Length Final (mm)	Shrinkage, *S* (%)	Mass Initial (g)	Mass Final (g)	% Change (--)
*x*	19.82	17.15	13.5	6.042	6.020	−0.37
*y*	19.72	17.02	13.7	6.054	6.028	−0.43
*z*	19.94	17.09	14.3	6.130	6.104	−0.43

**Table 5:** Summary of experimental density measurements.

Specimen	Density (g/cm^3^)	Relative Density (%)
*x*	7.72	97.1
*y*	7.76	97.6
*z*	7.69	96.7

**Table 6:** PSO-optimized parameters.

Parameter	Unit	*y*-Oriented	*z*-Oriented
Surface energy, $\gamma_{s}$	J/m^2^	1.42	1.30
Grain growth critical density, ${\;\rho }_{c}$	--	0.96	0.96
Grain growth pre exponential factor, $K_{0}$	μm^3^/s	4.45 × 10^3^	3.39 × 10^1^
Pre-exponential factor, $A_{\delta }$	Pa·s·K^−1^	1 × 10^−18^	1 × 10^−18^
Pre-exponential factor, $A_{\gamma }$	Pa·s·K^−1^	1.00	1.71
Activation energy for ferrite, $Q_{\delta }$	J/mol	709.3 × 10^3^	732.5 × 10^3^
Activation energy for austenite, $Q_{\gamma }$	J/mol	193.1 × 10^3^	193.1 × 10^3^
Transition temperature, $T_{trans}$	°C	1025	1014
Fitting parameter, p	--	17.1	15.6
Fitting parameter, λ	--	1.32	1.22
Initial relative density, $\rho_{0}$	%	62.6	61.7

## Data Availability

The original contributions presented in this study are included in the article. Further inquiries can be directed to the corresponding author.
